# An *In Vitro-In Vivo* Evaluation of the Antiproliferative and Antiangiogenic Effect of Flavone Apigenin against SK-MEL-24 Human Melanoma Cell Line

**DOI:** 10.1155/2021/5552664

**Published:** 2021-06-21

**Authors:** Alexandra Ghiƫu, Ioana Zinuca Pavel, Stefana Avram, Brigitta Kis, Daliana Minda, Cristina Adriana Dehelean, Valentina Buda, Roxana Folescu, Corina Danciu

**Affiliations:** ^1^Department of Pharmacognosy, “Victor Babeș” University of Medicine and Pharmacy Timișoara, Romania, Eftimie Murgu Sq. No. 2, 300041 Timișoara, Romania; ^2^Research Center for Pharmaco-Toxicological Evaluation, “Victor Babeș” University of Medicine and Pharmacy Timișoara, Romania, Eftimie Murgu Sq. No. 2, 300041 Timișoara, Romania; ^3^Department of Toxicology, “Victor Babeș” University of Medicine and Pharmacy Timișoara, Romania, Eftimie Murgu Sq. No. 2, 300041 Timișoara, Romania; ^4^Department of Pharmacology and Clinical Pharmacy, “Victor Babeș” University of Medicine and Pharmacy Timișoara, Romania, Eftimie Murgu Sq. No. 2, 300041 Timișoara, Romania; ^5^Department of Balneology, Medical Recovery and Rheumatology, “Victor Babeș” University of Medicine and Pharmacy Timișoara, Romania, Eftimie Murgu Sq. No. 2, 300041 Timișoara, Romania

## Abstract

One of the most important class of natural compounds with successful preclinical results in the management of cancer is the flavonoids. Due to the plethora of biological activities, apigenin (4′,5,7 trihydroxyflavone) is a main representant of the flavone subclass. Although the antiproliferative and antiangiogenic effects of apigenin were studied on a significant number of human and murine melanoma cell lines, in order to complete the data existing in the literature, the aim of this study is to evaluate the *in vitro* effect of apigenin on SK-MEL-24 human melanoma cell line as well as *in vivo* on tumor angiogenesis using the aforementioned cell line on the chorioallantoic membrane assay. Results have shown that in the range of tested doses, the phytocompound presents significant antiproliferative, cytotoxic, and antimigratory potential at 30 *μ*M, respectively, 60 *μ*M. Moreover, the phytocompound in both tested concentrations limited melanoma cell growth and migration and induced a reduced angiogenic reaction limiting melanoma cell development.

## 1. Introduction

From the earliest times, different types of plant extracts represented an important option for the treatment and/or prevention of a wide range of pathologies. From the past to the present, herbs have been used as teas, powders, different types of extracts, or purified active phytocompounds included in various pharmaceutical formulations [1]. According to World Health Organization, about 88% of member states are using traditional and alternative medicine [2]. Recently, modern techniques have allowed the isolation and/or synthesis/semisynthesis in the laboratory of a large number of pharmacologically active phytocompounds. The benefits of natural molecules are known from centuries, and this approach is expanding because of the significant capacity of phytocompounds—alone or in a synergistic way as found in extracts—to prevent or to treat different pathologies. To this aspect is added the fact that administered correctly, respecting the doses and the time interval, the vast majority of natural compounds have limited side effects. Up to date the main focus of the researchers is to identify and to establish the bioactive compounds' chemical structure and also their mechanism of action and toxicological impact [3] Since Plant Kingdom is an inexhaustible source of new bioactive molecules, an increased number of new molecules used in current well-established protocols in medicine are provided from natural compounds [1].

The variety of bioactive compounds is represented by different classes of phytocompounds including polyphenols, flavonoids, anthocyanins, saponins, triterpenes, tannins, alkaloids, glycosides, gums, resins, and volatile oils [4].

Many of these classes of phytochemicals have demonstrated to possess *in vitro* antiproliferative, proapoptotic, cytotoxic activity on different cancer cell lines, or/and anticancer activity in different experimental animal models [5–9]. Moreover, clinical trials that evaluate natural compounds have been conducted and are in progress [10–16].

One of the most important class of natural compounds with successful preclinical results in the management of cancer is the flavonoids. This polyphenolic family has a common structure of phenyl-benzo-piran, and depending on the oxidation degree, the nature of the substituents attached to C2/C3, and the hydroxylation pattern of the nucleus, the flavonoids are divided into different subclasses, of which the most important are flavanols, flavanones, flavonols, flavones, isoflavones, anthocyanidins, chalcones, aurones, and catechines [17].

The main interest of this current study is on the flavone apigenin (API), also known as 4′,5,7 trihydroxyflavone and characterized by C_15_H_10_O_5_ chemical structure. API is well distributed in a variety of fruits, vegetables, and medicinal plants. Significant sources of API can be found in chamomile, parsley, celery, spices, thyme, onion, tea, and wine [5].

Studies showed that the richest sources of API can be detected in dried chamomile (3,000-5,000 *μ*g/g), parsley (2,154.6 *μ*g/g), celery seed (786.5 *μ*g/g), Chinese celery (240 *μ*g/g), kumquats (218.7 *μ*g/g), celery (191 *μ*g/g), vine spinach (622 *μ*g/g), Mexican oregano (177.1 *μ*g/g), artichoke (74.8 *μ*g/g), juniper berries (72.6 *μ*g/g), and peppermint (53.9 *μ*g/g) [18].

API can be characterized by a variety of pharmacological properties. The phytocompound has proven to have anti-inflammatory and antioxidant properties, antibacterial, antiviral, antifungal, and antiparasitic activities [19]. It has been reported to decrease the level of LDL cholesterol, total cholesterol, and triglyceride and directly correlated to inhibit the process of atherosclerosis [20]. It has been showed that API has protective properties in myocardial injury [21] and is also effective in neuronal ischemia [19]. Salehi et al. have presented in their research paper that API improves renal disfunction and lowers the blood glucose in diabetes, attenuates the stress and depression symptoms, and has applicability in diseases like Alzheimer or amnesia by increasing the capacity of learning and memory skills [22] The authors also presented various clinical trials that monitored the beneficial effects of API supplementation in different pathologies (Alzheimer's disease, anxiety, depression, insomnia, and knee osteoarthritis) [22]. Zhou et al. have described that the phytocompound protects the endocrine system and exerts a positive role in osteoporosis by inhibiting the osteoclast and activation of osteoblasts. Also, one of the most studied properties is cytostatic and cytotoxic effects in different types of cancer [23].

The *in vitro* antitumoral effect of API has been studied and confirmed by many researchers on different cancer cell lines. Madunić et al. revealed that API (10 to 100 *μ*M) can induce morphological changes in MDA-MB-231 human breast cancer cell line in a time- and dose-dependent manner. Moreover, using the Annexin V/FITC assay, they have shown that API (20, 40, and 100 *μ*M) promotes the induction of late apoptotic death in MCF-7 cells. On the other hand, only the highest tested concentrations (60 and 100 *μ*M) triggered the same mode of death in MDA-MB-231 cells [24]. Seo et al. demonstrated that the phytocompound at different concentrations (10, 20, 40, and 80 *μ*M) influenced the inhibition of human prostate cancer cell line (PC3 and LNCap) proliferation after induction of apoptosis which was possible through increased p21 pathway and decreased PLK-1 expression as a result of apoptotic cell death [25]. In their study, Bai et al. proved that API could selectively inhibit different tumor cells. In the screened interval of concentrations (20–100 *μ*M), the phytocompound induced apoptosis of MCF-7 breast cancer cells, while the same concentration caused only a little damage on MCF-10A normal cells. The authors also indicated that API (80 *μ*M) induces early apoptotic events (from 1.1% to 18.6%). Moreover, the highest tested concentration (100 *μ*M) after an incubation period of 24 h induced a significant increase in late apoptosis and necrosis for MCF-7 cells (from 1.6% to 17.2%) [26]. Lindenmeyer et al. showed that treatment with this natural phytocompound (22.8-45.5 *μ*M) strongly inhibits the human MDA-MB-231 breast cancer cell invasion, migration, and also proliferation, events correlated to cell cycle arrest in the G2/M phase [27]. Since API has generated a great deal of interest, in a recent study, Liu et al. have demonstrated that this compound inhibits the proliferation of HeLa cervical cancer cell line with an IC_50_ = 47.26 *μ*M. The mechanism is based on inducing apoptosis and cell cycle arrest at G0/G1 phase. The authors reported that the cell proliferation is mainly related to the signalling pathways PI3K/AKT (intracellular signal transduction pathway) and PTEN (natural inhibitor). In this case, the PTEN/PI3K/AKT pathway was inhibited in a concentration-dependent manner, and cell migration was also impeded via matrix metalloproteinases 2 and 9 [28]. Up to date, Chen et al. demonstrated the anticancer activity of API using human HT-29 colon cancer cell line *in vitro* and also *in vivo* in xenografted mice models. Their results indicate that API at 20, 40, and 80 *μ*M *in vitro* and 35 mg/kg *in vivo* inhibits the proliferation of cancer cells and the growth of xenografted tumors. The same study reports the inhibition of some signalling pathways which play an essential role in the proliferation of cancer cells, namely, p-mTOR, p-PI3K, and p-AKT expressions in HT-29 cells concentration-dependently [29].

Nowadays, cancer is a pathology that affects people worldwide and is one of the main cause of death. One of the most aggressive types of cancer is represented by melanoma. The particularity of this type of skin cancer is given by a big metastatic capacity. The development of melanoma is linked also to the UV exposure because of the damages occurred on the DNA. One of the most representative classes of natural compounds with ability to absorb UV radiation is the flavonoids [30].

Researchers like Xu et al. showed that API has the capacity to affect cell proliferation (API 60 *μ*M-24h) and also has proapoptotic properties (API 30 *μ*M-24 h) on melanoma cell lines (A375, A2058, and RPMI-7951) [31]. Other groups have proven that API inhibited cell proliferation (API 40–160 *μ*M-48h) on A375 and C8161 melanoma cell lines, affected the capacity of cell migration and invasion (40 *μ*M-72h), and had proapoptotic effect (40 and 100 *μ*M-24h) when tested *in vitro* [32]. Das et al. demonstrated that API inhibited cell proliferation (20-250 *μ*g/mL-24h) and increased the number of cells in early and late apoptosis by damaging the normal function of the mitochondria (20, 50, and 80 *μ*g/mL-24 h) [33]. Chan et al. have noticed the antitumoral effect of API (10 and 20 *μ*M) on A431 squamous cell carcinoma by inducing cell cycle arrest in phase G2/M [34].

Tumor formation, development, progression, and metastasis require an activated angiogenesis process. Thus, targeting angiogenesis is included in the current anticancer therapeutic strategies. Natural compounds are being intensively researched as safer alternatives with multiple targeting potential. API was reported as an angiogenesis inhibitor in several types of cancers [35]. Still, the *in vivo* efficacy of API as antiangiogenic in melanoma was less investigated.

Regarding the fact that melanoma is one of the most aggressive type of skin cancer, many studies approached this topic. The researchers have proven the antiproliferative effect of API (10-50 *μ*M-24 h, 48 h, in a dose-dependent manner) on B16F10 murine cell melanoma line [36]. The antiproliferative and cytotoxic effect of API have been demonstrated also by testing this phytocompound (30 and 60 *μ*M-72h) on B164A5 murine melanoma cell line. The obtained results were in a dose-dependent manner [37]. Cao et al. have showed that API (150 mg/kg) has the ability to inhibit metastasis on B16F10 murine melanoma cell line. Moreover, in case of human melanoma cell lines A375, 518A2, and G361, API (40 *μ*M) inhibited in a dose-dependent manner the expression levels of STAT3 [38].

Malignant melanoma is one of the most aggressive forms among all types of cancer, and while significant advances in therapeutic management have improved melanoma patient care, there are still major clinical challenges such as drug resistance and recurrence to be overcome [39] API was investigated here, considering the promising anticancer activity by targeting several signalling pathways, next to limited secondary effects that were previously reported [32, 37, 38]. In previous studies, we established that API elicited in a dose-dependent way antimigratory and proapoptotic effects on different melanoma cells, on A375 human melanoma cell line [37], and on B164A5 murine melanoma cells [40].

Given the high impact of nanotechnologies, future perspectives could involve the evaluation of nanoformulations that contain API, a direction that could improve the biocompatibility and enlarge the spectrum of biological activities [41]. Some formulations that contain API already showed an improvement in solubility, bioavailability, and antioxidant properties [42]. These include nanosuspensions of API, nanocrystals of API, and API-loaded carbon nanotubes. API-loaded nanoparticles proved to reduce inflammation and fibrosis *in vitro* in chronic pancreatitis, and bovine serum albumin nanoparticles with API had a beneficial effect in lung injury. Nanoencapsulation of API in different nanoparticles improved the antitumor effect of API in various types of cancers [42]. Further *in vivo* and clinical studies are needed in order to establish the potential of nanoformulations that contain API in different pathologies.

Although the antiproliferative and antiangiogenic effects of API were studied on a significant number of human and murine melanoma cell lines, the intention of this study was to enrich the existing knowledge on the melanoma potential of API by exploring the impact of the compound on SK-MEL-24 human metastatic melanoma cell line, investigating *in vitro*, the cytotoxicity, viability, and antimigration effects, and *in vivo* on the chick chorioallantoic membrane, the antiangiogenic, and anti-invasion potential. Given the aggressive form of melanoma, we chose a human melanoma cell line derived from metastatic site, the lymph node.

## 2. Materials and Methods

### 2.1. Cell Culture

The cell line SK-MEL-24 human melanoma (ATCC® HTB-71) was acquired from the American Type Culture Collection (ATCC). Cells were cultured in Eagle's Minimum Essential Medium (EMEM; ATCC) supplemented with 15% fetal bovine serum (FBS; Sigma-Aldrich, Germany) and 1% penicillin/streptomycin mixture (Pen/Strep, 10,000 IU/mL; Sigma-Aldrich, Germany). Cells were maintained under standard conditions: 37°C and a humidified atmosphere of 5% CO_2_ in a Steri-Cycle i160 incubator (Thermo Fisher Scientific, USA).

### 2.2. MTT Assay

The effect of API (purchased from Sigma-Aldrich) on SK-MEL-24 human melanoma cells viability was evaluated by using the (3-(4,5-dimethylthiazol-2-yl)-2,5-diphenyltetrazolium bromide (MTT) assay. The method was performed as previously described [37]. In brief, a number of 1 × 10^4^ cells/well were seeded onto 96-well culture plates and allowed to adhere overnight. The next day, the cells were stimulated with different concentrations of API (0.3, 1, 3, 10, 30, and 60 *μ*M) and further incubated for 72 h. After 72 h of incubation, a volume of 10 *μ*L of 5 mg/mL MTT solution from the MTT kit (Sigma-Aldrich) was added in each well and incubated for an additional 3 h. The formed formazan crystals were dissolved by adding 100 *μ*L/well of lysis solution provided in the MTT kit. The cells treated with the solvent dimethyl sulfoxide (DMSO, Sigma-Aldrich) were used as the control group. The absorbance was spectrophotometrically analyzed at 570 nm with a microplate reader (BioRad, xMark Microplate Spectrophotometer).

### 2.3. Evaluation of the Cytotoxic Potential by Lactate Dehydrogenase (LDH) Assay

The cytotoxic rate of API at the highest tested concentrations (30 and 60 *μ*M) was evaluated by the quantification of the LDH amount released into the medium following cellular membrane damage. In order to perform the LDH assay (Cytotoxicity detection kit, 11644793001, Roche), a number of 5 × 10^3^ cells/well were seeded onto 96-well culture plates and allowed to adhere overnight. The next day, the cells were stimulated with the highest tested concentrations of API (30 and 60 *μ*M) and further incubated for 72 h. After the incubation period, a volume of 100 *μ*L was transferred from each well into a 96-well culture plate and mixed with 100 *μ*L of reaction mixture (obtained according to the manufacturer's instructions) and incubated for 30 min at room temperature.

The amount of LDH leakage in the medium was determined by measuring the absorbance at two wavelengths (490 nm and 680 nm) using a microplate reader (BioRad, xMark Microplate Spectrophotometer). In order to determine the spontaneous and maximum release of LDH, untreated cells (low control) and cells treated with 1% (*v*/*v*) Triton- X100 (high control) were used.

### 2.4. Scratch Assay

In order to assess SK-MEL-24 cell invasion capacity after stimulation with the highest tested API concentrations (30 and 60 *μ*M), a scratch assay was performed. Briefly, a number of 2 × 10^5^ cells/well were plated onto 12-well culture plates until a confluent cell monolayer was reached. Afterwards, a sterile pipette tip was used in order to draw a gap in the middle of each well. All the cells and cellular debris that detached following the procedure were gently washed with phosphate buffer saline (PBS). Further, the cells were stimulated with API (30 and 60 *μ*M). Pictures of the scratch area were taken at 0 h and 24 h at a magnification of 10x by means of an inverted microscope (Olympus IX73) equipped with DP74 camera (Olympus, Tokyo, Japan). For analyzing the cell growth, the cellSense Dimension software was used.

The following formula was used in order to calculate the scratch closure rate [43]:

(1) $$\begin{array}{c} \text{Scratch closure rate}=\left[\frac{A_{t0}-A_{t}}{A_{t}}\right]*100, \end{array}$$

where *A*_*t*0_ is the scratch area at time 0 h and *A*_*t*_ is the scratch area at 24 h.

### 2.5. Chorioallantoic Membrane Assay (CAM)

The CAM assay was used to investigate the effect of API on the angiogenic process under tumor development, using SK-MEL-24 human melanoma cells. This *in vivo* protocol is widely used to study neovascularisation in a tumor microenvironment and the potential inhibitors of new vessel formation. Using a slightly modified technique developed by Ribatti et al. [44], fertilized hen (Gallus gallus domesticus) eggs were disinfected and incubated at 37°C and 50% humidity. The protocol includes the removal of 3-4 mL of albumen on the third day of incubation, followed by cutting a window on the upper side of the eggs on the following day.

SK-MEL-24 melanoma cells cultured according to the above-described protocol were inoculated on top of the developing CAM on day 10 of incubation (day 0, 0 h) [45]. After detaching the cells from the culture plate by trypsinization, they were cleansed and resuspended in the culture medium until reaching the final concentration of 10^4^/5 *μ*L. A volume of 5 *μ*L of the SK-MEL-24 cell suspension was inoculated inside a plastic ring previously placed on each CAM. API in concentration of 30 *μ*M and 60 *μ*M, next to DMSO 0.5% as solvent control, was administered daily inside the melanoma cells-containing plastic rings. All specimens were monitored *in ovo*, and representative images were registered by means of a stereomicroscope (Discovery 8 Stereomicroscope, Zeiss) connected to digital camera Axiocam 105 color; software AxioVision SE64. Rel. 4.9.1 Software (Göttingen, Germany) and the images were processed by the Zeiss ZEN software, ImageJ (Image J Version 1.50e https://imagej.nih.gov/ij/index.html), and GIMP (GIMP v 2.8 GNU Image Manipulation Program https://www.gimp.org/).

### 2.6. Statistical Analysis

The data obtained in the *in vitro* section was analyzed using GraphPad Prism 5.0 (GraphPad Software, CA, USA). Comparison among groups was performed by means of One-way ANOVA test followed by Dunnett's multiple comparison posttest (^∗∗∗^*p* < 0.001 vs. control).

## 3. Results

### 3.1. MTT Assay

**Figure 1:**
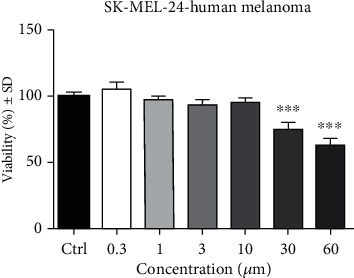
SK-MEL-24 human melanoma cell viability after 72 h stimulation with API (0.3, 1, 3, 10, 30, and 60 *μ*M). Results are expressed as cell viability percentage (%) related to control cells. Comparison among groups was performed by means of One-way ANOVA test followed by Dunnett's multiple comparison posttest (^∗∗∗^*p* < 0.001 vs. control).

The MTT assay illustrates the cell viability percentage of the human melanoma SK-MEL-24 cells. The effect of API was assessed on SK-MEL-24 cells after a 72 h stimulation period and compared to control.  Figure 1 depicts the effect of API (0.3, 1, 3, 10, 30, and 60 *μ*M) on melanoma cell viability. Obtained results indicate that in the range of tested concentrations, there is a decrease in tumor cell viability with the most significant reduction noticed at the two highest used concentrations (for 30 *μ*M, tumor cell viability was 75.08 ± 5.5% vs. control, and for 60 *μ*M, tumor cell viability was 62.9 ± 5.4% vs. control).

### 3.2. Assessment of the Cytotoxic Potential by means of LDH Release

**Figure 2:**
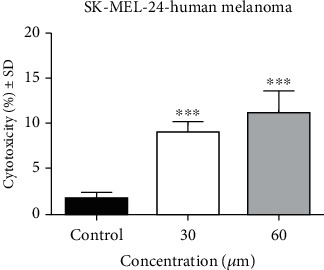
The cytotoxic effect of API (30 and 60 *μ*M) on SK-MEL-24 human melanoma cells after a stimulation period of 72 h. The results are expressed as cytotoxicity percentage (%) related to the control cells. Comparison among groups was performed by means of One-way ANOVA test followed by Dunnett's multiple comparison posttest (^∗∗∗^*p* < 0.001 vs. control).

Together with proliferation, cell cytotoxicity assays are frequently employed for drug screening, including natural molecules in order to detect whether the test compounds have effects. The cytotoxic potential of API determined by LDH assay is represented in Figure 2. The two highest concentrations of API (30 and 60 *μ*M) were selected due to the fact that the percent of viable cells observed through MTT assay was significantly lower following the stimulation. As shown in the graphic, a significant dose-dependent cytotoxic effect was provoked by API. At 30 *μ*M, the cytotoxic rate was 9 ± 1.1% vs. control (1.6 ± 0.7%), whereas at the highest dose tested, 60 *μ*M the cytotoxic rate was slightly higher, 11.1 ± 2.4% vs. control.

### 3.3. Scratch Assay

**Figure 3:**
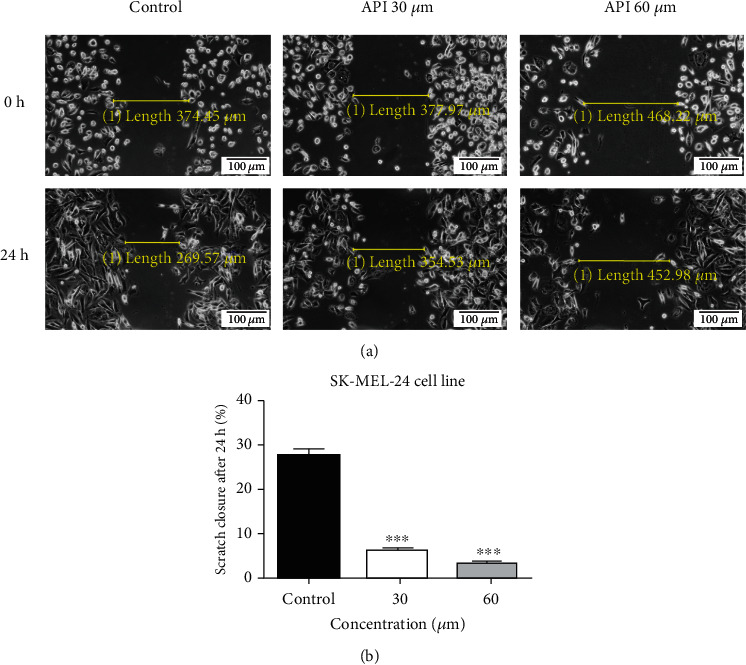
(a) The effect of API (30 and 60 *μ*M) on SK-MEL-24 human melanoma cell migration capacity. Images were taken by light microscopy at 10x magnification. Melanoma cell migration was monitored by imaging the scratch line initially and at 24 h poststimulation. (b) The bar graphs are expressed as percentage of scratch closure after 24 h compared to the initial surface (0 h). One-way ANOVA test and Dunnett's multiple comparison posttest were used for comparison among groups (^∗∗∗^*p* < 0.001 vs. control).

It has been already demonstrated that migration of cancer cells is a prerequisite for tumor cell invasion and metastasis, a totally unwanted event, antimigratory potential being a desirable property for an anticancer agent. The antimigratory potential of API at the highest tested concentrations (30 and 60 *μ*M) on SK-MEL-24 melanoma cells was determined by means of scratch assay.  Figure 3 shows that API elicited an inhibition of tumor cells migration in a dose-dependent manner.  Figure 3(a) depicts the effect of API at 0 h and 24 h, respectively, poststimulation. At the highest concentration tested, a slight change in cell shape can be noticed—some of them showed round shape and started to detached.  Figure 3(b) presents the scratch closure rate for the two concentrations used compared to control. At 30 *μ*M, API produced a scratch closure rate of only 6.2% and for 60 *μ*M an even lower one, namely, 3.4%. This effect indicates that API reduced melanoma cell migration.

### 3.4. API Effects on SK-MEL-24 Angiogenesis Using the CAM Assay

**Figure 4:**
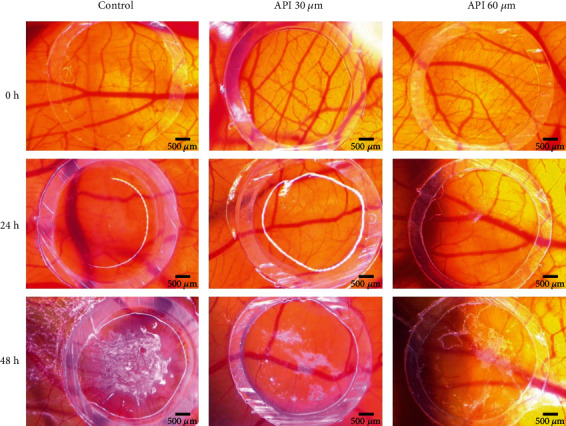
The antiangiogenic effect of API (30 and 60 *μ*M) using a SK-MEL-24 tumor CAM assay. Representative stereomicroscope images were registered initially (0 h), after 24 h, and 48 h after inoculation. Scale bars represent 500 *μ*m.

It is very well known that an activated angiogenesis process is required for tumor formation, development, progression, and metastasis. To assess the potential antiangiogenic effect of API in a melanoma microenvironment, we used the tumor CAM assay, with SK-MEL-24 human melanoma cells. Observing the control group, where only 0.5% DMSO was applied, the development of tumor cells was not affected, and cell area extended progressively. After 24 hours of incubation with the compound, only some compact tumor cell areas were noticed inside the application ring, while after 48 hours postinoculation, a large and compact tumor area covered most of the inner surface of the ring, and extended areas of tumor cells were present outside the ring. Blood vessels formed a densely network in a spokes wheel pattern towards the application ring (Figure 4).

API in both tested concentrations limited melanoma cell growth and migration and induced a reduced angiogenic reaction. As shown in Figure 4, after incubation with API for 48 h, tumor cell areas were limited, and only a few scattered cells were observed outside the application spot. The number of capillaries was decreased compared to the control sample; the vessels in peritumoral areas had a narrow aspect and showed a low branching profile. Minimal differences could be observed between the two tested concentrations of API, with the higher concentration inducing a slightly higher decrease in tumor cell development and attenuation of neovascularisation.

## 4. Discussion

Nowadays, a large number of studies including *in vitro*, *in vivo*, and clinical trials highlight the fact that API is a potential therapeutic agent used for the management of several diseases [46].

Regarding the anticancer activities, in recent years, API has gained a significant importance as a health promoting agent due to its reduced toxicity and due to the fact that this phytocompound does not affect healthy cells but is active on various cancer cell lines [47].

The major findings of the present study indicate that in the screened conditions API reduced SK-MEL-24 melanoma cell migration and viability in a dose-dependent manner, the most significant results being obtained at 30 and 60 *μ*M. Furthermore, a cytotoxic effect was obtained at 30 and 60 *μ*M determined by LDH release. To the best of our knowledge, this is the first study that presents the effects of API on SK-MEL-24 human metastatic melanoma cells. Our data are in accordance with the scientific literature where it is indicated that this flavone has an antiproliferative effect against different melanoma cell lines [32, 37, 40]. Our research group has previously established that API (0.3–60 *μ*M) is a potential antiproliferative and proapoptotic agent against A375 human melanoma cell line, with an IC_50_ of 33.02 *μ*M [37]. Following the same thought, Woo et al. investigated the effect of API (25, 50, 75, and 100 *μ*M) for 24 h on human melanoma cells (A375P and A375SM). Using *in vitro* assays, this research group has shown that the cell viability was decreased, and the expression of Bax, p53, PARP, and cleaved caspase-9 increased in a dose-dependent manner [48]. Using A2058 and A375 melanoma cell lines, Hasnat et al. pointed out that API treatment (10, 20, and 50 *μ*M) inhibited the growth of melanoma cells. Moreover, API treatment increased apoptotic factors such as caspase-3, cleaved poly (ADP-ribose) polymerase, and also modulation of FAK/ERK and integrin signalling pathways [49]. The phytocompound also exhibited proapoptotic properties on A375, A2058, and RPMI-7951 melanoma cells. The researchers showed that API (30 *μ*M) presented inhibitory effects on the expression levels of PD-L1 and stimulation of IFN-*γ* [31]. Another study investigated the effects of this natural molecule on B16F10 melanoma cells. The research group showed that API in the dose ranges of 10-50 *μ*M, after incubation periods of 24 and 48 h, presented significant increase of cell proportion in the G0/G1, S, and G2/M phases followed by a significant decrease in the G0/G1 phase [36].

Deepening the experimental line, Woo et al. transplanted the A375SM human melanoma cells in nude mice which were treated intraperitoneally with API (25 and 50 mg/kg). Results showed that the tumor size and weight were significantly decreased in a dose-dependent manner in the API treated groups when compared with the control group [48]. Cao et al. demonstrated the antimetastatic effect of API *in vivo* using C57BL/6 mice. Results showed that in the murine melanoma B16F10 cell line, API (150 mg/kg) presented antimetastatic activity by suppressing STAT3 phosphorylation and downregulating MMP-2, MMP-9, and VEGF pathways [38]. Xu et al. investigated API effects *in vivo* on melanoma xenograft mouse models using the B16-F10 melanoma cell line. This group suggested that API treatment (150 mg/kg) significantly suppressed the growth of B16-F10 xenograft tumors and regulated the sensitivity of tumor to immune killing by increased the abundance of CD4+ and CD8+ T cells [31].

Various research paper showed the *in vitro* antitumor effect of API on several types of cancer cells, including MDA-MB-231 and MCF-7 human breast cancer cell lines [24, 26, 27], PC3 and LNCap human prostate cancer [25], HeLa cervical cancer cell line [28], and HT-29 colon cancer cell line [29].

Other *in vivo* studies also pointed out that intraperitoneally administered API can attenuate the tumor growth in B16-BL6 murine melanoma metastasis model (50, 25, and 12.5 mg/kg) [50], Lewis lung carcinoma (LLC) and DHDK-12 colonic cancer line (50 mg/kg/day) [51], pancreatic cancer PCS (50 mg, once daily) [52], human hepatocellular carcinoma cells (HepG2-fLuc and HepG2-GFP cells) (0.5 mg/kg/2 days) [53], renal cell carcinoma (RCC) cells (30 mg/kg every 3 days for 21 days) [54], colorectal adenocarcinoma DLD1 and SW480 cells (20 mg/kg once a week) [55], human ovarian cancer A2780 cells (5 mg/kg body weight) [56], human MDA-MB-231 breast cancer cells (5 or 25 mg·kg^−1^ every day for 8 weeks) [57], and colorectal cancer (20 mg/kg) [58].

ROS (reactive oxygen species) have been reported as an important regulator for mitochondrial function [59]. Furthermore, the overproduction of ROS can damage cellular components such as proteins, lipids, and DNA and also plays a significant role in apoptosis induction or can induce cell death. Along the same line of thought, Bai et al. showed that API treatment increases the ROS level which led to apoptosis in the API-treated MCF-7 cells [26].

In different cancer cells, high levels of ROS (reactive oxygen species) can appear from increased metabolic activity and mitochondrial function and also from increased activity of oxidases, cyclooxygenases, lipoxygenases, and cellular receptor signalling or through oncogene activity. Furthermore, the overproduction of ROS can damage cellular components such as proteins, lipids, and DNA and also plays a significant role in apoptosis induction or can induce cell death [60, 61]. Along the same line of thought, Bai et al. showed that API treatment increases the ROS level which leads to apoptosis in the API-treated MCF-7 cells [26].

Tumor necrosis factor- (TNF-) related apoptosis-inducing ligand (TRAIL) is an encouraging anticancer agent which induces apoptotic cell death in numerous cell lines by creating a complex with death receptors. This complex is able to trigger the activation of the death-inducing signalling complex and also the caspase signalling cascade [62]. Taken together these findings, researchers reported that API upregulates ROS generation; however, the ROS inhibitors (glutathione and N-acetyl-L-cysteine) present a moderate increase of API/TRAIL-induced apoptosis by a slight increase of ROS generation. The results were compared with rapamycin (autophagy inducer) which improved apoptosis by a slight increase of ROS generation [63]. The same conclusion was drawn by Shukla et al. In their approach, the research group indicates that API (10–80 *μ*M) induces apoptosis in human 22Rv1 prostate cancer cells. They have shown that this event is initiated by ROS-dependent disruption of the mitochondrial membrane potential by transcriptional-dependent and -independent p53 pathways in a dose- and time-dependent manner. Moreover, API treatment increased the level of cleaved caspase-3 and modified Bax and Bcl-2 protein levels, whereon favor apoptosis [64].

There is clear evidence that API exert antiproliferative and proapoptotic effects by multiple and complex mechanism. The key signalling pathways by which API employ its anticancer properties are highlighted mainly by the upregulated levels of caspase [65], Bax, and PARP levels [66]; therefore decrease Bcl-2 and Mcl-1 levels [33, 44, 67] decreased the cytochrome C in the mitochondrial fraction [67]; inhibited NF-*κ*B activation [68]; suppressed the expression of cyclin B1, Cdc2, and Cdc25c; induced PARP cleavage; and induced LC3-II [69].

In order to gain more details regarding the effect of API on the metastatic human melanoma cell line, we performed the tumor chorioallantoic membrane assay. Considered as valuable intermediate *in vivo* biological tool as a preliminary assay to rodent *in vivo* animal assays, the model has several advantages, including costs, time, simplicity, and reproducibility [45] API was investigated as an antiangiogenic agent with potential benefits in hindering cancer progression and metastasis for several types of cancer by targeting several signalling pathways [70]. API has proven to be involved in the inhibition of angiogenesis by suppressing expression of VEGF and HIF-1*α* [71], inactivation of ERK1/2 [72], inhibition of STAT3 signalling, and decreasing the levels of the MMP-2 and MMP-9 expression [38]. Less data is available regarding the effects of API on the angiogenesis process associated to human melanoma microenvironment. Our previous investigation on API effects on an A375 melanoma model showed the potential effect of decreasing angiogenic and tumor progression [37]. The results obtained here on an SK-MEL-24 melanoma model using the chorioallantoic membrane assay come to emphasise that treatment with 30 and 60 *μ*M API induces a reduced neovascularisation next to limiting melanoma cell growth and migration.

API at 30 and 60 *μ*M reduced SK-MEL-24 melanoma cell viability and migration and produced slight changes in melanoma cell morphology at 24 h poststimulation. In addition, in the tumor chorioallantoic membrane, API decreased tumor cell development. Once more, API showed an antitumor effect on the melanoma cells. In the upcoming studies, we will address the effect of API *in vivo*, using experimental animal models. Furthermore, for a targeted drug delivery in melanoma, various nanoformulations are taken into consideration.

## 5. Conclusion

Results from the present study show that API in the tested dose ranges presents *in vitro* antiproliferative properties against SK-MEL-24 human metastatic melanoma cell line. At 30 and 60 *μ*M, API elicited a dose-dependent cytotoxic effect and showed antimigratory potential. Moreover, treatment with 30 and 60 *μ*M API using the tumor chorioallantoic membrane assay induces a reduced neovascularisation next to limiting melanoma cell development; hence, API could be considered as a promising antiangiogenic agent in malignant melanoma. Overall, API proved to possess antitumor effect on SK-MEL-24 melanoma cells, an effect that can be assessed in future studies. Moreover, due to the impact of nanotechnologies, future directions could involve the design of nanoformulations such as apigenin-conjugated silver or gold nanoparticles in order to increase the bioavailability of this flavone for *in vivo* experimental models and also to expand the spectrum of biological activities [41]. Thus, API can become a valuable tool in the management of chronic diseases such as cardiovascular diseases, diabetes, and neurological diseases. As indicated in various studies [41, 42], the use of natural compounds or of various nanoformulations that include them could be a beneficial strategy, benefitting from higher biocompatibility, biodegradability, and biomimetic features. Further clinical trials involving API could be performed as a possible strategy for the management of melanoma. Since the number of articles involving the study of nanotechnology in humans is lower compared to nonnanotherapies, an evaluation of API in such a manner could be of real interest.

## Data Availability

All data used to support the findings of this study are included within the article.

## Abbreviations

BAX: Bcl-2-associated X protein
Bcl-2: B-cell lymphoma 2
Cdc2 and Cdc25c: Cell division cycle
P53: Cellular tumor antigen
PARP: Poly-ADP ribose polymerase
FAK/ERK: Adhesion-mediated focal adhesion kinase
IFN-*γ*: Interferon gamma
STAT3: Signal transducer and activator of transcription 3
MMP-2, MMP-9: Matrix metallopeptidase
VEGF: Vascular endothelial growth factor
mTOR: Mammalian target of rapamycin
NF-*κ*B: Nuclear factor kappa
p-PI3K: Phosphatidylinositol 3-kinase
p-AKT: Protein kinase B
LC3-II: Cytosolic form of LC3
TNF: Tumor necrosis factor
TRAIL: Tumor necrosis factor- (TNF-) related apoptosis-inducing ligand.
